# Interspecific trait variability and plasticity of the Baltic Sea phytoplankton species along a salinity gradient

**DOI:** 10.1093/plankt/fbaf015

**Published:** 2025-04-24

**Authors:** Iris D S Orizar, Aleksandra M Lewandowska

**Affiliations:** Tvärminne Zoological Station, Faculty of Biological and Environmental Sciences, University of Helsinki, J.A. Palmenin 260, 10900 Hanko, Finland; Tvärminne Zoological Station, Faculty of Biological and Environmental Sciences, University of Helsinki, J.A. Palmenin 260, 10900 Hanko, Finland

**Keywords:** salinity, phytoplankton resource use efficiency, trait-based, Baltic Sea, trait plasticity

## Abstract

In the face of changing climate and global water cycle, the plastic response of phytoplankton species to salinity fluctuations is increasingly important. This study used a multivariate approach to determine interspecific trait variability and plasticity of 10 Baltic Sea phytoplankton species along the salinity gradient. Phytoplankton species representing a broad range of sizes and taxonomic groups were grown at six salinity conditions (0, 5, 15, 20, 30 and 35 psu), and 15 different traits were measured at the end of the experiment. Results showed species-specific salinity preferences. Nutrient uptake and resource use efficiency (RUE) explained interspecific trait variability among the species. Variability in nutrient uptake reflected species-specific differences in cell size. RUE and cellular elemental content were the most plastic traits across the salinity gradient and did not scale with cell size. Interestingly, low trait plasticity did not always translate into low biomass production, as a diatom *Phaeodactylum tricornutum* exemplified. As expected, the salinity range between 5 and 20 psu was optimal for most phytoplankton species, corresponding to the brackish Baltic Sea where they were isolated. Many species survive in salinities above this range, but not in freshwater, which can have consequences for the plankton community functioning with predicted Baltic Sea freshening.

## INTRODUCTION

The high diversity of phytoplankton communities allowed them to colonize various aquatic ecosystems, providing important ecosystem functions and services. Phytoplankton richness and evenness have been shown to affect their biomass production capacity (Fargione *et al.*, 2007), and resource use efficiency (RUE) (Ye *et al.*, 2019; Otero *et al.*, 2020). The diversity of the phytoplankton community varies naturally through time and space and is highly affected by multiple abiotic factors, such as temperature (Schabhüttl *et al.*, 2013; Lewandowska *et al.*, 2014; Righetti *et al.*, 2019), pH (Pedersen and Hansen, 2003), nutrients (Lehtinen *et al.*, 2017) and salinity (Olli *et al*., 2019, 2023). However, our understanding of the effects of salinity changes on the biodiversity–ecosystem functioning relationships in phytoplankton is still incomplete.

Salinity is a critical environmental factor determining the distribution of many aquatic organisms. Sea surface salinity is affected by global and local drivers. The global water cycle affects sea surface salinity through evaporation (increases salinity) and precipitation (decreases salinity). Locally, salinity is affected by river run-off, tides and ice formation. A global salinity shift is predicted to occur due to climate change, wherein saltier ocean regions become more saline, and fresher ocean regions become fresher (Durack and Wijffels, 2010; Durack *et al.*, 2012). In addition, anthropogenic activities, such as road salting and watershed modifications, locally affect sea surface salinity (Wolanski and Elliot, 2015; Smyth and Elliott, 2016). These global and local drivers have varying impacts on the salinity of different aquatic environments, affecting species composition and phytoplankton community structure.

Phytoplankton species can be classified based on their tolerance to salinity change: stenohaline species have a narrow salinity tolerance range, while euryhaline species have a broad salinity tolerance range. From the evolutionary perspective, phytoplankton had to adapt to freshwater conditions after moving from a hypersaline aquatic environment, where the first phytoplankton species, presumably cyanobacteria, appeared (Knauth, 2005). The persistence of semi-permeable cellular membranes across all living organisms is strong evidence of the first living cells’ adaptation to a saline environment.

Increased or decreased salinity conditions pose different problems to phytoplankton cells. Algal cells exposed to fresher conditions need to find a solution to the increased water flow movement towards the cell, increasing the turgor pressure, while algal cells exposed to increased salinity conditions must prevent water loss from the cell, resulting in shrinkage. Exposure to hypoosmotic shock can affect photosynthetic activity (Campbell *et al.*, 2019) and lipid production (Vrana *et al*, 2022) of algal cells, while exposure to hyperosmotic stress has been shown to affect polyphosphate hydrolysis (Weiss *et al.*, 1991; Leitão *et al.*, 1995). Organisms that allow internal fluid concentrations to vary with the environment are called osmoconformers, relying on behavioral responses such as avoiding stressful environmental change by moving away. Osmoregulators, on the other hand, can maintain their internal fluid concentration at an acceptable level despite the environmental changes (Smyth and Elliott, 2016).

Salinity also affects phytoplankton buoyancy, influencing their ability to compete for the limited space in the euphotic zone. Objects are more buoyant in seawater than in freshwater due to higher seawater density. Thus, phytoplankton species, especially from freshwater habitats, adopted various approaches to improve their buoyancy, such as: (i) having organelle (e.g. flagella, cilia and gas vesicles) to improve mobility (Walsby, 1994); (ii) producing positively buoyant compounds (e.g. lipids and ammonium compounds) to remain afloat (Lund, 1959; Boyd and Gradmann, 2002); and (iii) taking shape and size (including cell aggregation, chain and colony formation) that reduces the sinking rate (Lovecchio *et al.*, 2019). Some phytoplankton species exposed to salinity changes undergo morphological modification, changing cell size and shape toward a higher surface area-to-volume ratio to increase buoyancy (Vrieling *et al.*, 2007; Aizdaicher and Markina, 2010; Leterme *et al.*, 2010; Trobajo *et al.*, 2011; Fu *et al.*, 2022). The ability of phytoplankton species to cope with salinity changes depends on their plasticity and genotypic variability (Sjöqvist and Kremp, 2016; Argyle *et al.*, 2021; Orizar and Lewandowska, 2022), but exposure to salinity conditions beyond optimal range can have sublethal to lethal effects. In the Baltic Sea and the Chesapeake Bay, the minimum phytoplankton diversity was observed between 7 and 9 psu (Olli *et al.*, 2019), which is a result of the filtering effect of salinity against freshwater and marine phytoplankton species. Phytoplankton within the transitional conditions are considered brackish species (Remane, 1934), tolerating frequent salinity changes. The unique salinity conditions of the Baltic Sea present a challenge to phytoplankton, which could affect the interspecific variability of the phytoplankton community, further affecting their ecosystem functioning. The salinity of the Baltic Sea is maintained by evaporation (199 ± 3 km^3^ year^−1^), precipitation (256 ± 6 km^3^ year^−1^) and water flow from land (476 ± 17 km^3^ year^−1^) (Boulahia *et al.*, 2022), but regional climate models predict precipitation to intensify, resulting in salinity decline. The Baltic Sea freshening may lead to a reduction of phytoplankton diversity with negative consequences for ecosystem functioning. For example, the Baltic Sea freshening and temperature increase can favor harmful algae by providing more suitable environmental conditions for their growth while providing suboptimal to lethal conditions for competing non-harmful species (Gobler, 2020; Trainer *et al.*, 2020).

We used a multivariate trait-based approach to characterize interspecific variability of the Baltic Sea phytoplankton species and their plastic responses along the salinity gradient. Specifically, we asked if the response of the Baltic Sea phytoplankton species to changing salinity conditions is consistent among taxonomic groups, and which traits are affected the most. Can the Baltic Sea species express trait plasticity when grown under different salinity conditions, considering their life history and adaptation to brackish environments? Or did the exposure to low salinity conditions result in the loss of trait plasticity required to tolerate other higher/lower salinity conditions? We hypothesized that: (i) the overall trait variation is reduced toward extreme salinity conditions (Stefanidou *et al.*, 2018; Olli *et al.*, 2023), and (ii) species will show contrasting strategies for coping with salinity stress that will be expressed by different set of traits (Orizar and Lewandowska, 2022). In our analysis, we focused on traits describing nutrient uptake and allocation (e.g. RUE and cellular elemental content), which are expected to respond to salinity changes (Davison and Reed, 1985; Kirst, 1989). Those traits are typically correlated with cell size (Litchman and Klausmeier, 2008; Finkel *et al.*, 2010; Kerimoglu *et al.*, 2012; Marañón *et al.*, 2013), therefore we selected species that represent a broad size range.

## MATERIALS AND METHODS

### Phytoplankton cultures and experimental design

We selected 10 phytoplankton species from the Finnish Marine Research Infrastructure (FINMARI) algae Culture Collection hosted at the Tvärminne Zoological Station, Finland. All strains were isolated from the Baltic Sea (Table I) at salinities 5–6 psu. The cultures were maintained under laboratory conditions (salinity ~6 psu, temperature = 4/16°C, light = 16:8 light–dark cycle).

**Table I:** The study included the location and year of isolation of the 10 phytoplankton species

Species	Strain	Year collected	Location	Cell length (μm)	Biovolume (μm^3^ ± sd)
*P. tricornutum*	TV335	≤1992	Tvärminne	15–17^a^	115 ± 51^g^
*D. tenuis*	DTTV B5	2008	Tvärminne	40–80^a^	567 ± 304^g^
*L. fissa*	GFF 1101	2011	Åland/Föglö	22–49^b^	9039 ± 2452^g^
*K. veneficum*	KVDAN 31	2013	Raseborg/Danskog	11–15^c^	~700–800^h^
*K. foliaceum*	KFF 0901	2009	Åland/Föglö	22–45^a^	96 503 ± 38 974^g^
*Rhodomonas marina*	CRYPTO 07B1	2007	Tvärminne	20^a^	382 ± 129^g^
*R. nottbecki*	CRYPTO 07B3	2007	Tvärminne	10–17^d^	No data
*D. lutheri*	TV 03	≤1992	Tvärminne	4–6^d^	37 ± 10^g^
*Monoraphidium* sp.	TV 70	≤1992	Tvärminne	2–182^f^	9 ± 4^g^
*Synechococcus* sp.	TV 65	≤1992	Tvärminne	1–3^a^	5 ± 3^g^

Note:

^a^ Olenina *et al.*, 2006

^b^ Moestrup *et al.*, 2014

^c^ Dai *et al.*, 2014

^d^ Majaneva *et al.*, 2014

^e^ Throndsen, 1997

^f^ Guiry and Guiry, 2020

^g^ Orizar *et al.*, 2024

^h^ Coyne *et al.*, 2021

The stock cultures were acclimated to the following conditions: (i) light = 130 μmol photon m^−2^ s^−1^ (16:8-h light–dark cycle); (ii) F/2 medium nutrient supply (NO_3_ = 882 μM, PO_4_ = 36 μM, Si = 106 μM, trace metals and vitamins); (iii) temperature = 16°C; (iv) and salinity = 6 psu, for at least four growth cycles.

The mono-algal experiments were conducted by growing each of the 10 phytoplankton strains under six salinity conditions: 0, 5, 15, 20, 30 and 35 psu. Sixty milliliters of the stock cultures were transferred to tissue culture flasks containing 540 mL fresh F/2 media at desired salinity levels (three replicates). The temperature and light conditions were kept the same as the acclimation period. The chlorophyll *a* (chl-*a*) fluorescence (a proxy for biomass) was measured with the Varian Cary Eclipse fluorescence (excitation: 430 nm, emission: 580 nm), and used for growth monitoring. The first monitoring was conducted 24 h after the experiment initiation and will be referred to as day 0. The fluorescence measurements were done at the same time of the day throughout the experiment. A salinity treatment was terminated once the stationary phase was reached or no sign of growth was observed for three consecutive monitoring days (6 days). Samples for microscopy, chl-*a*, particulate carbon (POC), nitrogen (PON), phosphorus (POP) and residual inorganic nutrient concentrations were collected after treatment termination.

### Analysis of the samples

Specific growth rates (μ) were calculated using chl-*a* fluorescence of the cultures at the beginning and end of the exponential growth phase following Guillard equation (Guillard, 1973). Fluorescence is only a proxy for cell density, as the experimental conditions might affect the cell’s chl-*a* concentration. Thus, observed changes in μ can also reflect variation in the cellular chl-*a* content. The specific growth rate of experimental units that did not show increased chl-*a* fluorescence during incubation was set to zero.

Samples for microscopy (100 ml) were preserved with acidic Lugol’s iodine solution. Phytoplankton cells were counted using DM IRB inverted microscope equipped with LASX software (Leica, Wetzlar, Germany) following Utermöhl method (Utermöhl, 1958) and HELCOM proceedings (Lund *et al.*, 1958; HELCOM, 2018). For *Synechococcus* sp., 200 μl 37% formaldehyde were added to 4 ml of the samples. The cell density was determined using BD Accuri C6 Plus Flow Cytometer.

The chl-*a* samples were collected by filtering cultures (maximum of 100 ml) on GF/F filters. The chl-*a* was extracted using 94% ethanol for 24 h in the dark, and fluorescence was measured using Varian Cary Eclipse fluorescence spectrophotometer. The total chl-*a* fluorescence was converted using chl-*a* standard curve and was normalized using cell density data to calculate the chl-*a* content cell^−1^.

POC/N and POP were collected on acid-washed, pre-combusted GF/F filters. For POC/N samples were dried at 60°C for 24 h and analyzed using an elemental analyzer (vario MicroCube, Elementar, Germany). POP samples were analyzed using the method developed by Koistinen *et al.* (Solorzano and Sharp, 1980; Koistinen *et al.*, 2017). The total POC, PON and POP concentrations were normalized using cell density to calculate cellular content.

Inorganic nutrient samples were collected from the residual of the cultures filtered through a cellulose acetate membrane syringe filter (pore size = 0.20 *μ*m). Residual concentrations of NO_x_ (NO_3_ + NO_2_), NH_4_, PO_4_ and Si were determined using a continuous flow autoanalyzer (AAII) following Hansen and Koroleff (2007). The net nutrient uptake rates (S) of NO_x_ (will be referred to as NO_3_ for the rest of the article) and PO_4_ were calculated using the following equation:

Equation 1. The net nutrient uptake rate


$$ S=\frac{N_{t0}-{N}_{t1}}{C\ast \Delta t} $$

where *N*_t0_ = *F*/2 media concentration (μM),

*N*_t1_ = residual nutrient concentration at the end of the incubation (μM),

*C* = cell density at the end of the incubation (cell ml^−1^),

*t* = days of incubation (day).

Uptake rates were set to zero when the calculation resulted in negative values.

RUE of nitrogen (RUE_N_) and phosphorus (RUE_P_) were calculated as log(POC/TN) and log(POC/TP), respectively, following a modified Olli *et al.* equation (Olli *et al.*, 2023). In these equations, POC is a particular organic carbon concentration in μg l^−1^ and represents realized phytoplankton productivity at the end of the incubation period. TN and TP are total nitrogen and total phosphorus concentrations (sums of dissolved inorganic, dissolved organic and particulate organic N and P, respectively) measured at the end of the incubation period using a continuous flow autoanalyzer (AAII). TN and TP represent potential productivity of the phytoplankton species.

### Statistical analysis

We used a principal component analysis (PCA) to reduce the dimensionality of the trait response of the species to salinity changes. Following Argyle *et al.* (2021), PCA of traits measured under 5 psu conditions were used to establish the basal trait-scape of the 10 species. This represents the interspecific trait variability under the salinity conditions where the stock cultures were maintained. An extended trait-scape was created from the results of PCA of the pooled trait data measured from all the salinity treatments (0, 5, 15, 20, 30 and 35 psu). Then, hierarchical cluster analysis of the principal components (HCPC) was performed on the extended trait-scape to investigate the clustering mechanisms along the salinity gradient and trait variation. Note that cell size was excluded from the PCA analysis, because the species were intentionally selected to represent a broad size range, and this would otherwise increase collinearity between traits.

Species plasticity was calculated following Argyle *et al.* (2021). The average distance between the species centroid and PC coordinates was used as a proxy for species plasticity along the salinity gradient. Interspecific variability per salinity condition was estimated by taking the average distance of the species centroid from the salinity centroid.

All statistical analyses and data visualization were done using R statistical software ver 4.2.2 (R Core Team, 2022). The *FactoMiner* package (Le *et al.*, 2008) was used to implement PCA and HCPC, and *factoextra* (Kassambara and Mundt, 2020) and ggplot2 (Wickham, 2016) were used to visualize the results.

## RESULTS

### Performance of the phytoplankton species along the salinity gradient

**Fig. 1:**
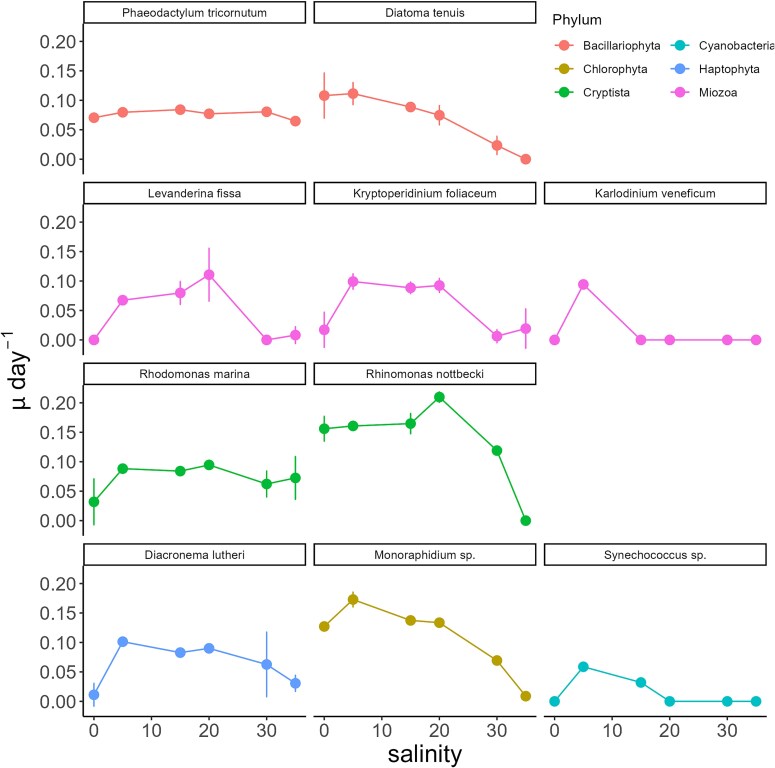
Growth rates of the 10 Baltic Sea phytoplankton species at different salinity conditions. Line color corresponds to the phylum of the species. Error bars = standard deviation from the mean (*n* = 3).

The growth rate was used to indicate species-specific salinity preferences. All 10 species grew well in 5 psu (Fig. 1). Only four species (*Phaeodactylum tricornutum*, *Diatoma tenuis*, *Rhinomonas nottbecki* and *Monoraphidium* sp.) showed significant growth under freshwater conditions (0 psu). Growth of three species (*D. tenuis*, *Monoraphidium* sp. and *Synechococcus* sp.) was negatively affected by salinity >5 psu. Most of the species showed a positive growth between 5 and 20 psu. The diatom *P. tricornutum* had the broadest salinity tolerance, while the dinoflagellate *Karlodinium veneficum* showed a specific salinity preference to 5 psu (Fig. 1).

### Standard trait-scape for the 10 phytoplankton species

**Fig. 2:**
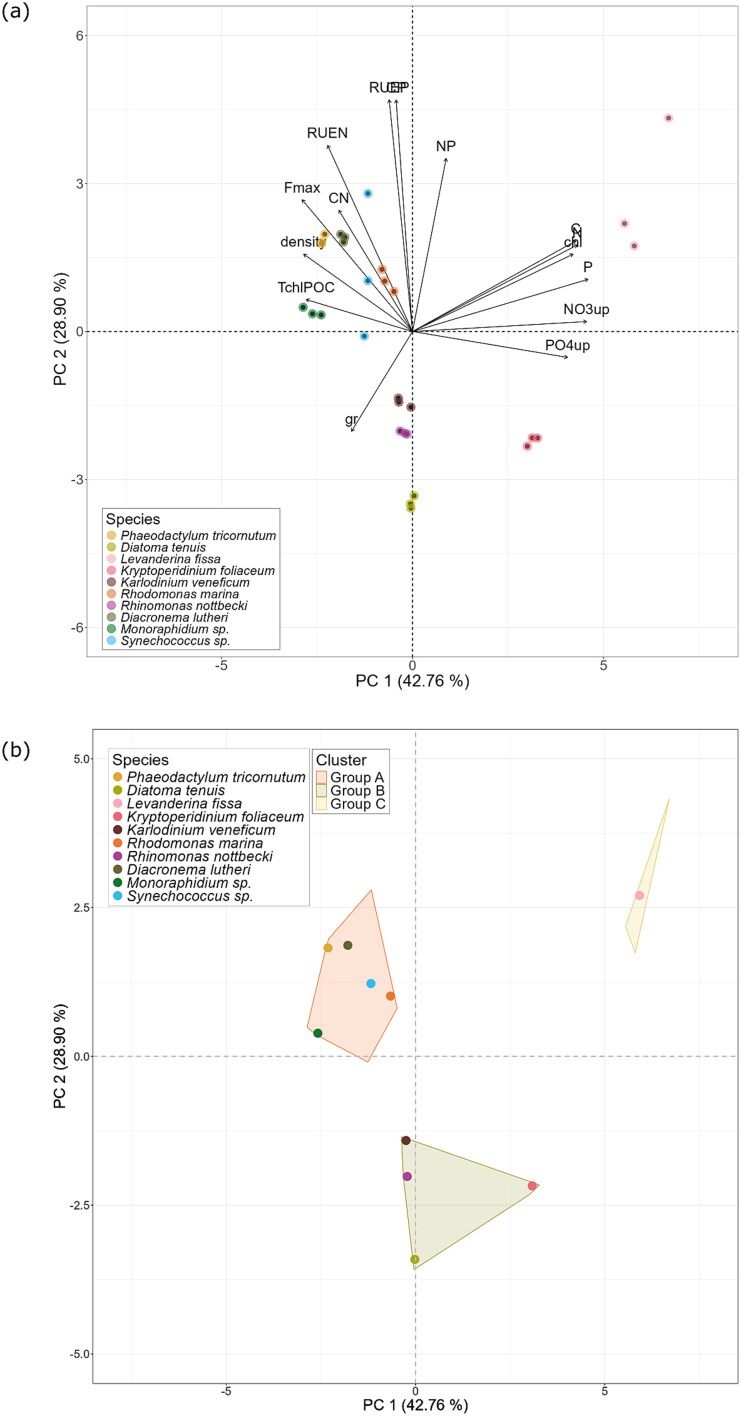
(**a**) Biplot of the first two principal components of the PCA on 15 phytoplankton traits measured from the 10 phytoplankton species grown under 5 psu. Each point represents one replicate. (**b**) Biplot showing the HCPC clustering of the species. Each point represents the species centroid (*n* = 3).

The standard trait-scape for the Baltic Sea strains was created from the principal components (PCs) of the traits measured at 5 psu. The first two PCs explained ~71% of the total trait variation (Fig. 2). Nitrate uptake rates and cellular PON/P content primarily described the interspecific variability among the species under the 5 psu condition (Table S1). These four traits showed a high correlation with each other (Fig. S1). On the other hand, RUE_p_ and C:P ratio described the interspecific trait variation along the second PC axis (Table S1).

HCPC grouped the 10 species into three clusters (Fig. 2b). Group A is characterized by high RUE and biomass production but low nutrient uptake rates. Group B is characterized by low RUE and biomass production. Finally, Group C, which only includes *Levanderina fissa*, is characterized by high nutrient uptake rates and cellular POC, PON and chl-*a* content. The dinoflagellates, *L. fissa* and *Kryptoperidinium foliaceum* had the highest nitrate uptake rates, while the diatom *D. tenuis* had the lowest nitrate uptake rates and RUE_P_ (Fig. S2).

### Interspecific trait plasticity along the salinity gradient

**Fig. 3:**
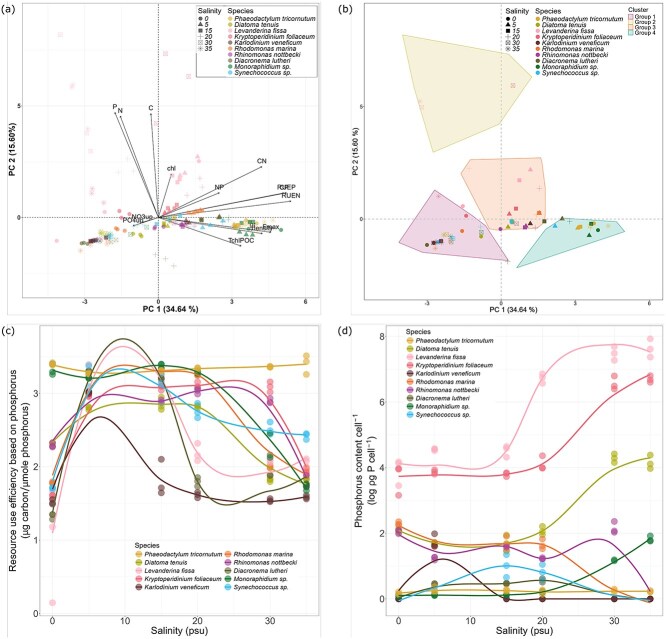
(**a**) Biplot of the first two principal components of the PCA on 15 phytoplankton traits measured for 10 phytoplankton species from the Baltic Sea grown at six salinity conditions (0, 5, 15, 20, 30 and 35 psu). Each point represents one replicate. (**b**) Biplot showing the HCPC clustering of the treatment (salinity × species). Each point represents the centroid of a treatment (*n* = 3). (**c**) RUEP, and (**d**) phosphorus content per cell by species for each salinity condition. Each dot is a replicate. Smoother line was generated using geom_smooth(method = loess) function from the ggplot2 package.

Interspecific trait plasticity was investigated using the PCA results of the phytoplankton traits measured from the 10 species grown at different salinity conditions (0, 5, 15, 20, 30 and 35 psu, Fig. 3). The first two PCs explained 50% of the trait variability among the treatments (salinity × species). The treatments had varying RUEs and C:P ratios (Table S2) along the first PC axis and cellular POC, PON and POP content along the second axis. RUE and C:P ratio, and POC, PON and POP were highly correlated (Fig. S3).

When considering specific traits that contributed the most to the variability along PC1 and PC2, the RUE_P_ was the lowest at 5 psu for most of the species, except *P. tricornutum* (Fig. 3c), which was characterized by low RUE_P_ in all treatments. Also, *Monoraphidium* sp. had low RUE_P_ under low salinity conditions where it grew the best (Fig. 1). Cellular P content (Fig. 3d) was, however, rather similar under different salinity conditions for most of the species, except *Monoraphidium* sp. and the two dinoflagellates *L. fissa* and *K. foliaceum*.

HCPC grouped the treatments into four groups (Fig. 3b). Group 1 is composed of species grown at 0 and 35 psu, characterized by low cellular POC and POP content. Group 2 is composed mainly of *L. fissa* and *K. foliaceum* grown under 30 and 35 psu, characterized by high cellular POC, PON and POP content. Group 3 is composed of all species grown at 5–20 psu, except *P. tricornutum* and *Monoraphidium* sp. Group 3 was characterized by high growth rate (*μ*), chl-*a* content and RUE. Group 4 comprises *P. tricornutum* and *Monoraphidium* sp. from all salinity conditions and is characterized by high biomass production, C:P ratio and RUE.

**Fig. 4:**
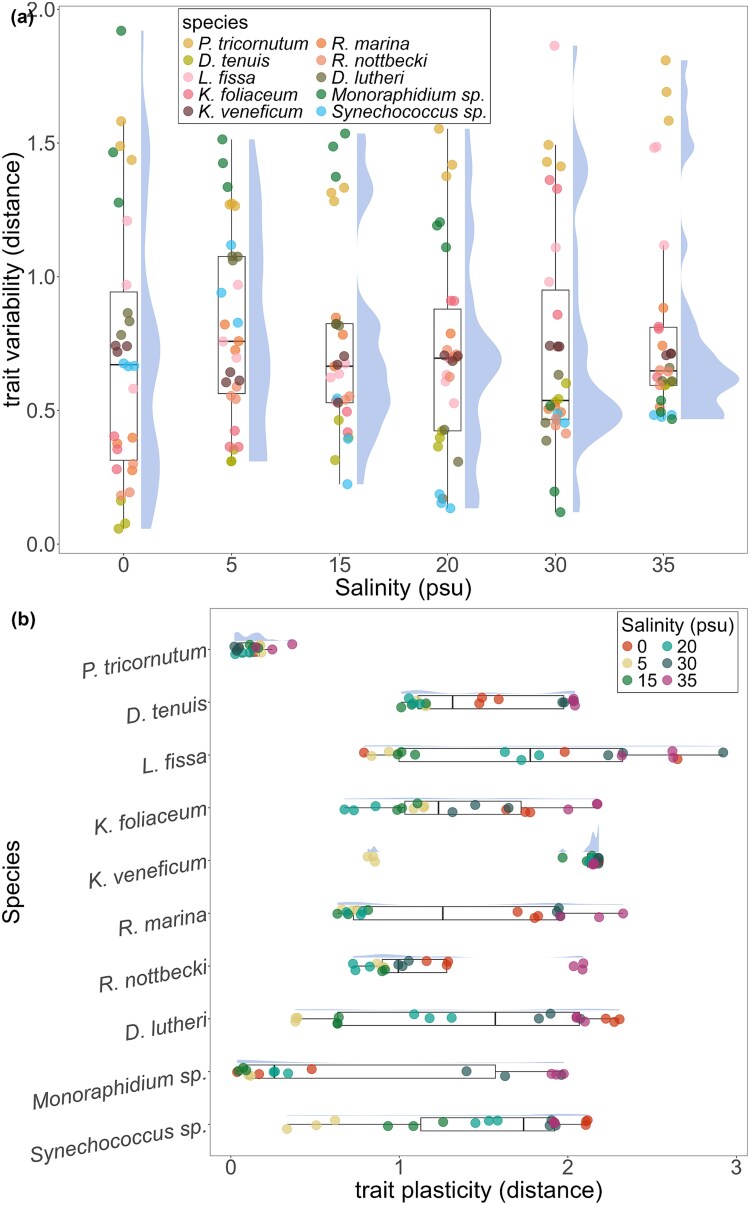
(**a**) Interspecific trait variation calculated as the distance between each replicate and the salinity centroid. Colors represent different species. (**b**) Species-specific trait plasticity calculated as the distance between each replicate and the species centroid. Colors represent different salinity treatments.

The distance between centroids was used to estimate interspecific trait plasticity among the 10 species along the salinity gradient (Fig. 4). The highest interspecific variation was at the 5 psu treatment (Fig. 4a), but there was no significant difference among the means (sum of sq = 0.005, *F* = 0.028, *P*-value = 0.87). The species showed varying trait plasticity along the salinity gradient (Fig. 4b), with *P. tricornutum* showing the lowest and *K. veneficum* the highest trait plasticity. *Synechococcus* sp., *Diacronema lutheri* and *D. tenuis* had similar trait plasticity along the salinity gradient, while *P. tricornutum* and *L. fissa/K. veneficum* had the highest dissimilarity in trait plasticity. Interestingly, species that had a narrow salinity preference but managed to grow beyond the preferred salinity conditions showed high overall trait plasticity, while *P. tricornutum,* which showed similar growth across the salinity gradient, had low overall plasticity. In addition, *K. veneficum* appears to have high overall trait plasticity, even though it only grew significantly under 5 psu.

## DISCUSSION

The Baltic Sea phytoplankton are exposed to brackish salinity conditions and pronounced salinity gradients. An analysis of phytoplankton *α* diversity in the Baltic Sea indicates seven as the lower limit of marine species salinity tolerance and nine as the upper tolerance limit for freshwater species (Olli *et al.*, 2019). Our results show that the Baltic Sea phytoplankton have species-specific salinity preferences that are not consistent within the phyla or the single traits. Most of the species had a positive growth rate between 5 and 20 psu corresponding to the brackish conditions of the Baltic Sea where they were isolated from, but some of them grew under freshwater conditions (Fig. 1). The freshwater native *Monoraphidium* sp. (Guiry and Guiry, 2020) was able to grow up to 30 psu. In contrast, *K. veneficum*, a Baltic Sea isolate of marine origin, grew only in 5 psu, although it has been known to form blooms in environments with a wide range of salinity conditions, such as the Chesapeake Bay (3–29 psu) (Li *et al.*, 2000,) and the Baltic Sea (Karlson *et al.*, 2021). A previous study has reported a slower growth rate of phytoplankton species (*Alexandrium ostenfeldii*) at lower salinity conditions (Martens *et al.*, 2016), which could indicate a reallocation of resources from cell division to responses to salinity changes such as production of osmolytes (hyperosmotic stress) or active removal of solutes (hypoosmotic stress). The observed positive growth rates of many marine-native species in this study suggest that the Baltic Sea is an incubator of unique phenotypes of phytoplankton species capable of overcoming the negative impact of salinity decrease, but potentially on the cost of fast growth. It is also likely that the phytoplankton species genetically adapted to the brackish conditions of the Baltic Sea and no longer pose marine characteristics, or that they adapted to the cultivation conditions (6 psu).

The results of the multivariate analysis imply a strong interspecific trait variability among the species (Fig. 2). Nutrient uptake rates were the main sources of interspecific variation under 5 psu conditions, wherein the bigger dinoflagellates had high uptake rates compared to the smaller species such as *Monoraphidium* sp. and *Synechococcus* sp. This is in line with earlier findings, which identified cell size as an important factor that determines the maximum nutrient uptake rate of phytoplankton species (Marañón, 2015). In contrast, RUE, which described the interspecific variability along the PC 2 axis, showed no direct size dependence (Fig. S4). *P. tricornutum, D. lutheri* and *L. fissa,* representing different size classes, phyla and HCPC groups (Fig. 2b), had similar RUE_P_ (Fig. S4). Phytoplankton cell size is known as a master trait correlated with other traits, such as nutrient uptake rates and Carbon:Nitrogen:Phosphorus (CNP) stoichiometry (Brown *et al.*, 2004; Litchman and Klausmeier, 2008; Weithoff and Beisner, 2019), but a size-dependent trade-off between nutrient uptake and metabolism has also been reported (Ward *et al.*, 2017). In our study we found a trade-off between RUE and growth rate (Fig. 2), suggesting that high RUE resulted in increased carbon storage in the cell rather than faster cell division.

The relationship between cell size and other traits becomes more complicated when considered in the context of environmental gradients such as salinity (Fig. 3). Salinity stress can restrict RUE and affect the elemental content of phytoplankton cells, e.g. by adding P-rich hydrophobic lipids to the cell wall to decrease water inflow when exposed to hypoosmotic pressure (Bemal and Anil, 2018; Antacli *et al.*, 2021). The PCA of the 15 traits measured along the salinity gradient (six salinity levels) suggests high interspecific trait plasticity of the Baltic Sea phytoplankton to salinity change. RUE (explaining PC 1) and cellular elemental content (explaining PC 2) were the most plastic traits (Fig. 3). The low growth rate and biomass production of Groups 1 and 2 suggest that 0, 30 and 35 psu were lethal to sublethal conditions for most of the Baltic Sea phytoplankton species. Although it should be considered that our growth rate estimate was based on fluorescence rather than cell density and can therefore reflect changes in cellular chl-*a* content with salinity to some extent (Fig. S7).

The clustering of multiple species grown between 5 and 20 psu conditions in Group 3 suggests that this is the optimal salinity range for the Baltic Sea species and does not elicit plastic trait response. Plastic response is more pronounced when the conditions are suboptimal, but not lethal to the species (DiGiacopo and Hua, 2020; Bond *et al.*, 2021). Interestingly, *P. tricornutum* and *Monoraphidium* sp., which were clustered in Group 4, had low trait plasticity along the salinity gradient (Fig. 4), with *P. tricornutum* showing overall broad salinity tolerance (Fig. 1). The N:P ratios of *P. tricornutum* suggest a change in nutrient acquisition strategy towards luxury uptake of P in response to changing salinity conditions (Fig. S5), resulting in an N:P ratio similar to the initial nutrient concentrations (Fig. S6). On the other hand, the nutrient uptake rates of *Monoraphidium* sp. increased with salinity, but the ratio of the particulate to dissolved inorganic nutrients decreased, suggesting reallocation of the resources from reproduction to cell maintenance, perhaps osmoregulation due to the exposure to a hyperosmotic environment, leading to a decrease in cell density with increasing salinity.

## CONCLUSIONS

As expected, the optimal salinity range for most of the Baltic Sea phytoplankton species is within the range of 5–20, which corresponds to the brackish conditions they have been living in. While many species have the potential to survive in salinities above this range, less can survive in freshwater. If the salinity of the Baltic Sea declines as predicted, many phytoplankton species might become locally extinct, with negative consequences for biodiversity and functioning of the Baltic Sea pelagic ecosystem. However, low phytoplankton trait plasticity does not necessarily result in low RUE and POC concentration, as exemplified by *P. tricornutum*. The ability of *P. tricornutum* (and other euryhaline species) to maintain a high growth rate and biomass production across a broad range of salinity conditions can be essential for the coastal community experiencing salinity fluctuations.

## Supplementary Material

Orizar_and_Lewandowska_Supplementary_fbaf015

## Data Availability

The raw data supporting the conclusion of this article will be made available by the authors, without undue reservation.
