# Supplementary material for: Interspecific trait variability and plasticity of the Baltic Sea phytoplankton species along a salinity gradient
Source: J Plankton Res. 2025 Apr 24;47(3):fbaf015. doi: 10.1093/plankt/fbaf015 (PMC12021263; doi:10.1093/plankt/fbaf015)
Supplement: Orizar_and_Lewandowska_Supplementary_fbaf015 [file orizar_and_lewandowska_supplementary_fbaf015.docx]

**Interspecific trait variability and plasticity of the Baltic Sea species along a salinity gradient**

**Orizar, I.D.S.^1^ and Lewandowska, A.K.^1^**

^1*^Tvärminne Zoological Station, Faculty of Biological and Environmental Sciences, University of Helsinki, Hanko, Finland

*corresponding author:

Iris D.S. Orizar

irisorizar@gmail.com

**Supplementary Figures**


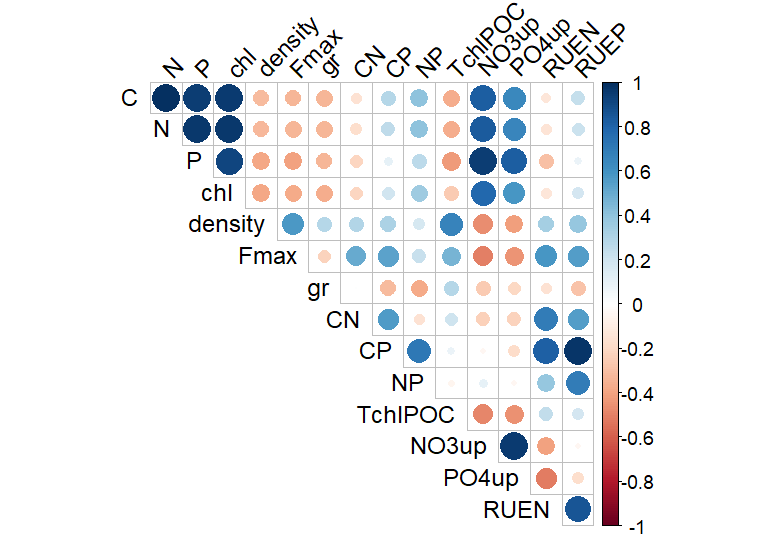


**Fig S1**. Correlation matrix of the 15 traits (see Table S2) measured at 5 psu treatment.


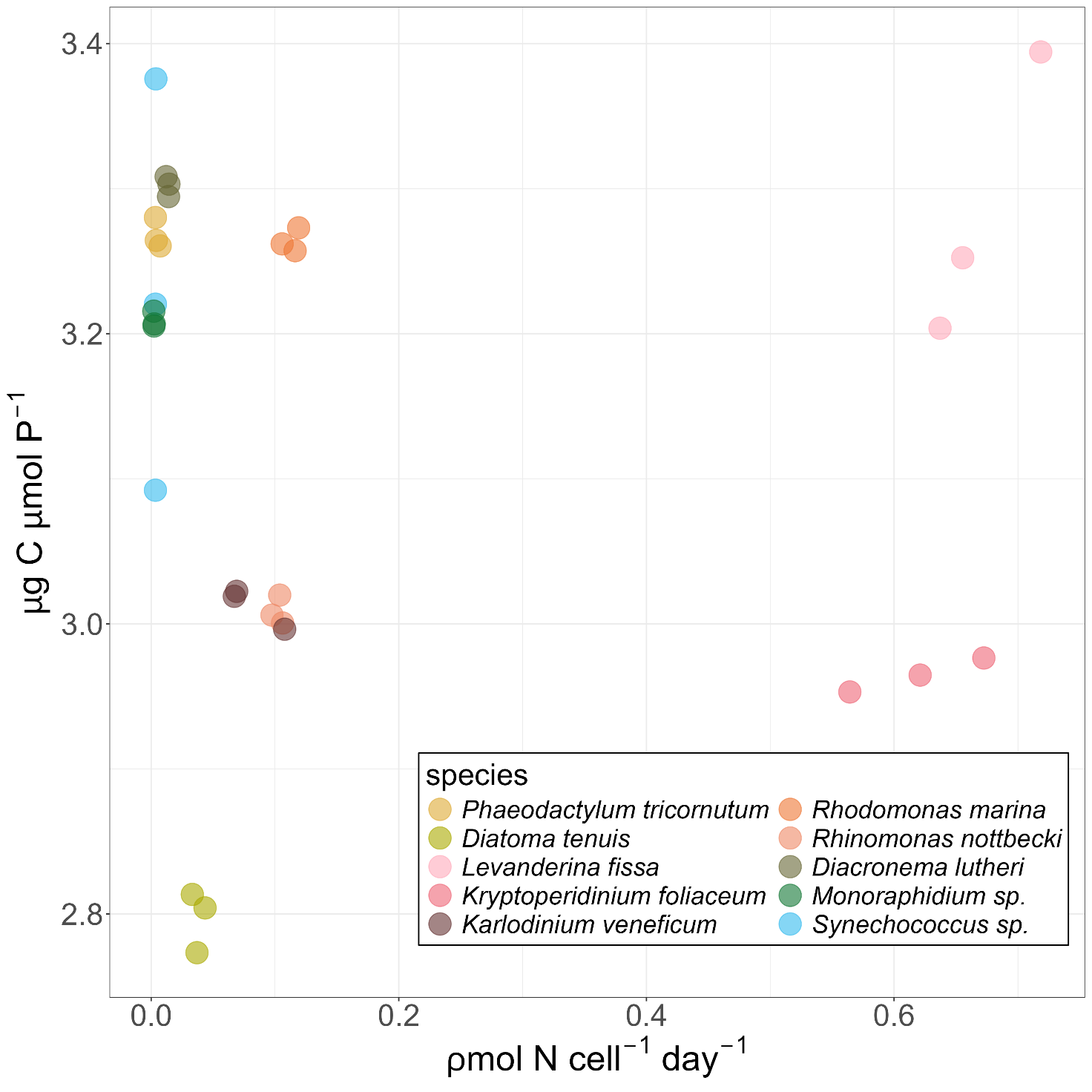


**Fig. S2.** Nitrate uptake rate vs RUE_P_ of the species under 5 psu conditions. Each point is one replicate.


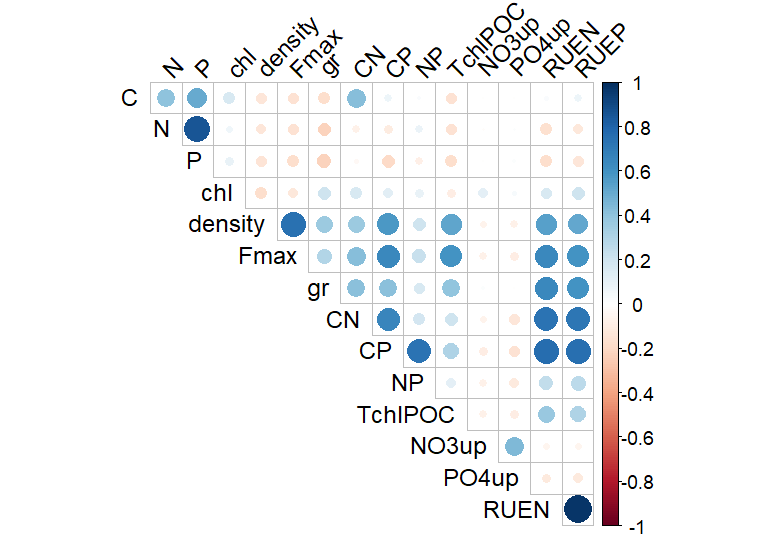


**Fig S3.** Correlation matrix of the 15 traits (see Table S3) measured at 6 psu.


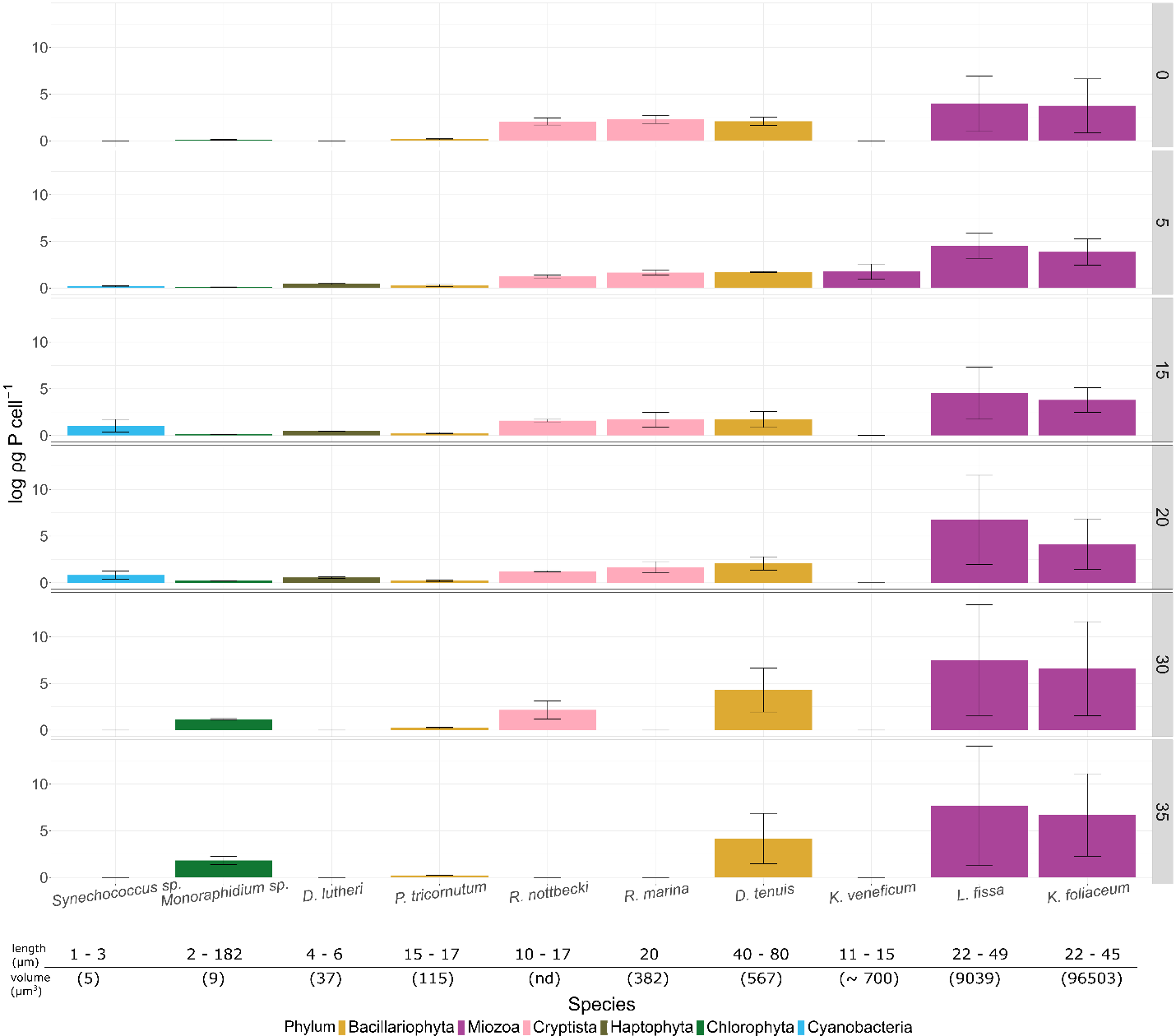


**Fig. S4.** Average RUE_p_ of each species grown at different salinity levels (n = 3). Color corresponds to the Phylum to which each species belongs. Error bars = standard deviation from the mean.


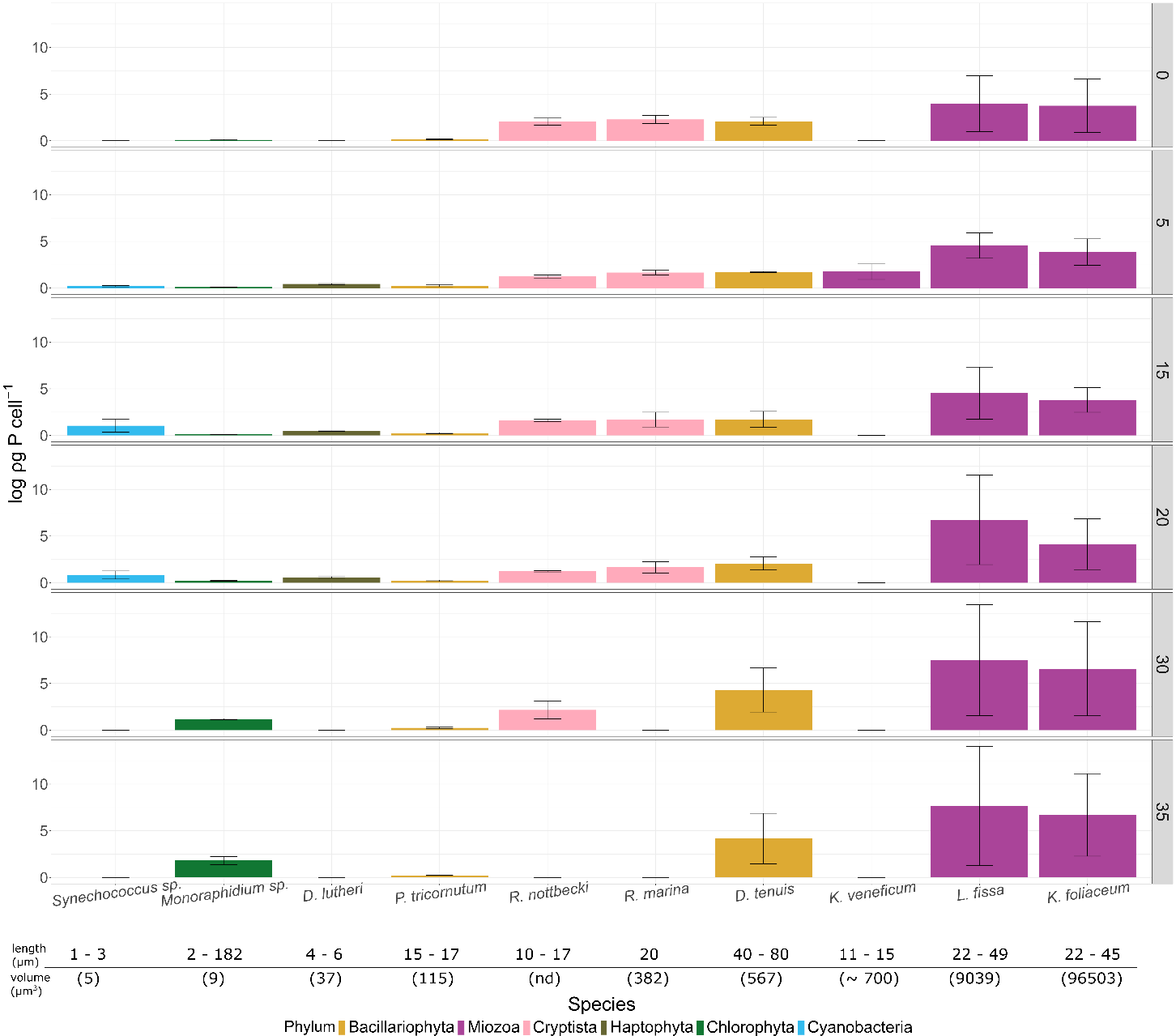


**Fig. S5.** Average phosphorus content cell^-1^ of each species grown at different salinity levels (n = 3). Colored corresponds to the Phylum to which each species belongs. Raw average phosphorus content cell^-1^ was transformed using the following formula: log(y + 1), where y is the raw value.


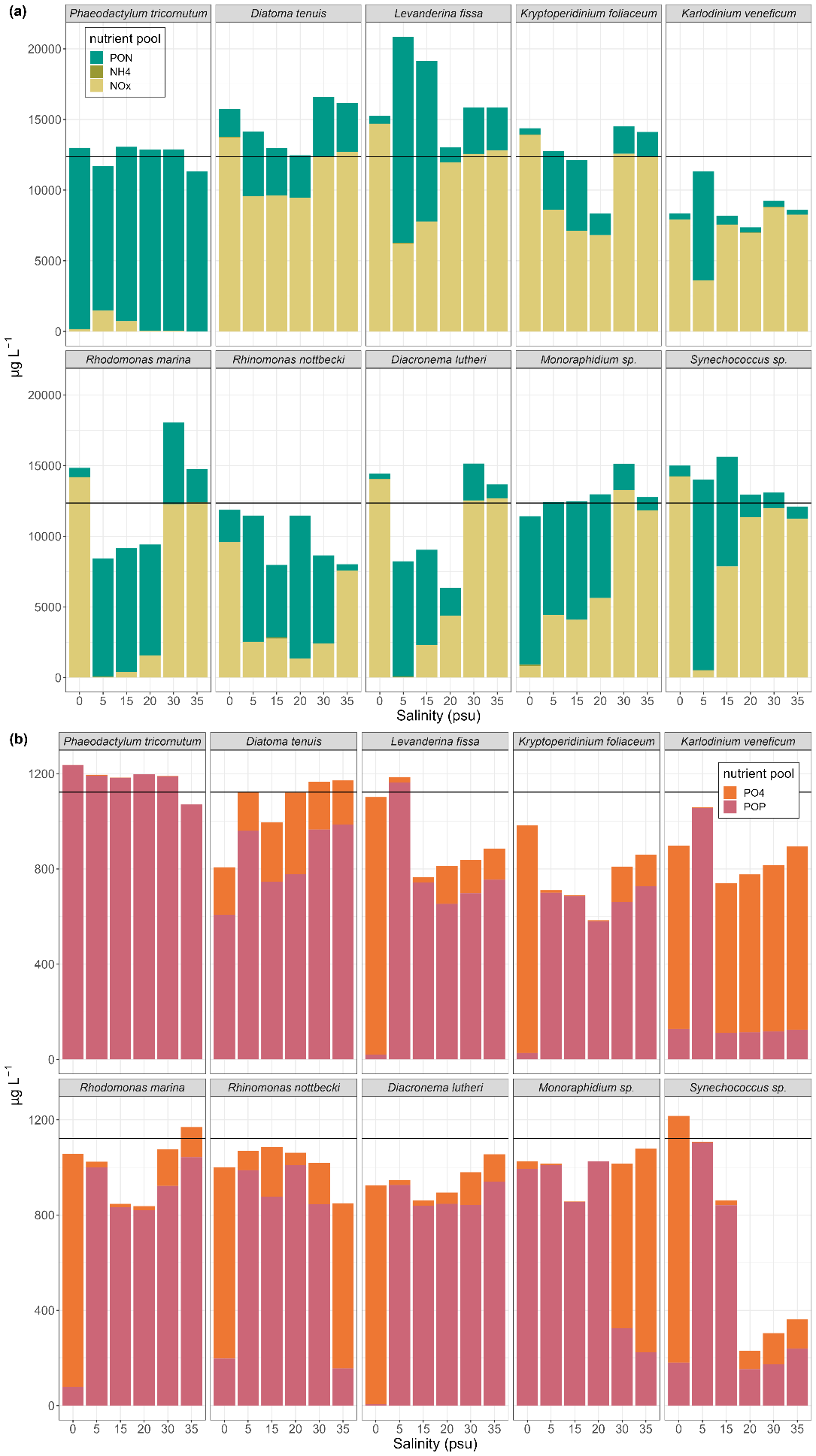


**Fig. S6.** Species-specific nitrogen (a) and phosphorus (b) pools (dissolved inorganic and particulate organic nutrients) along the salinity gradient (n = 3). Solid horizontal lines are nitrogen and phosphorus concentrations in the F/2 media.


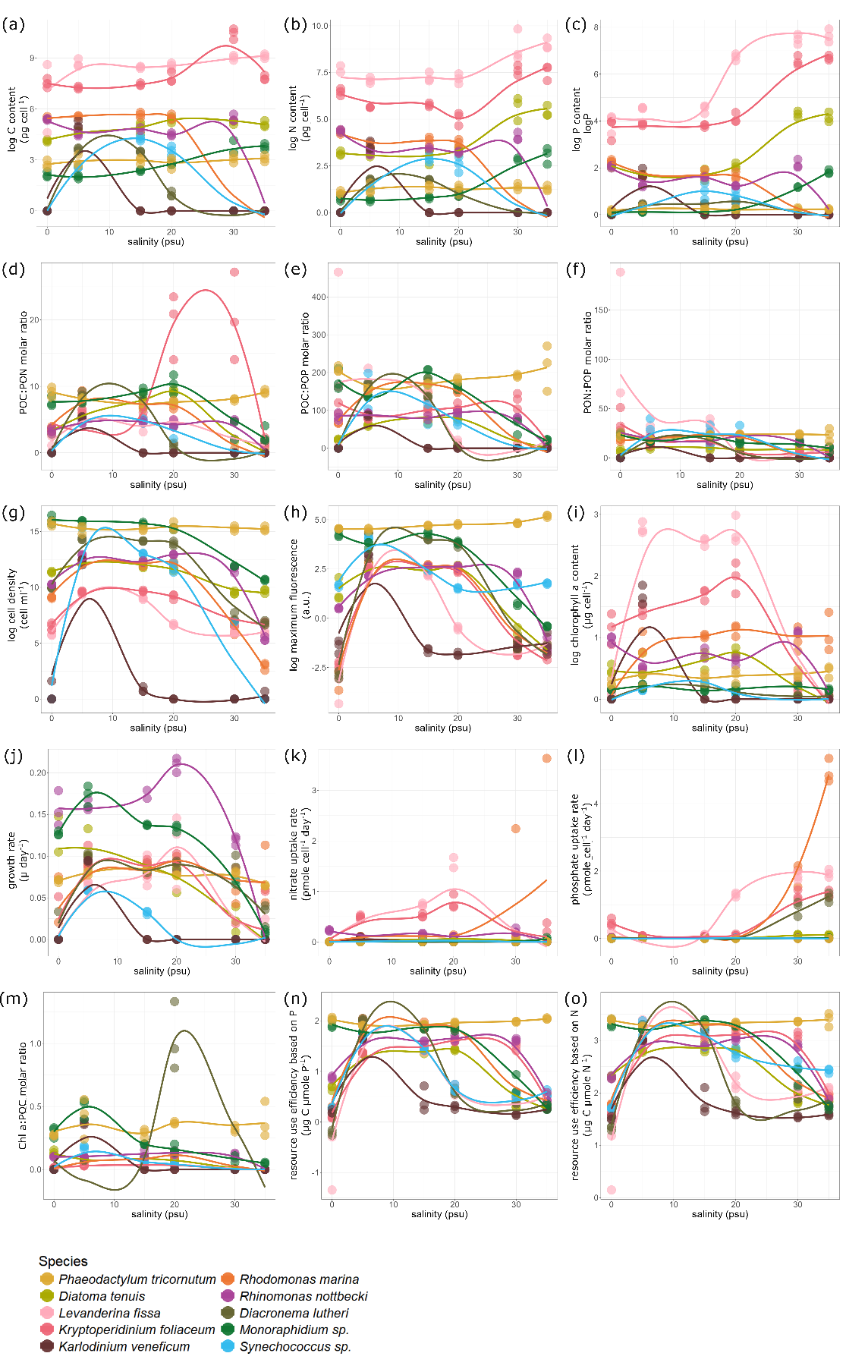


**Fig. S7.** Dot plots of the 15 traits (a – o) measured from each of the ten phytoplankton species grown at six salinity conditions. Smoother line were generated using *geom_smooth(method = ‘loess’)* function in the *ggplot2* package. Log-transformed traits for visualization was calculated following the formula: log(y + 1), where y is the raw value of the trait. (a – c) particulate organic carbon, nitrogen, and phosphorus, respectively, (d – f) POC:PON, POC:POP, and PON:POP molar ratio, (g) cell density, (h) maximum fluorescence (F_max_), (i) chlorophyll *a* content cell^-1^, (j) growth rate, (k) nitrate uptake rate, (l) phosphate uptake rate, (m) chl *a*:POC molar ratio, (n – o) resource use efficiency based on nitrogen and phosphorus, respectively.

**Supplementary Tables**

**Table S1**. PCA results of the 15 traits measured at 5 psu only.

|  |  | PC 1 | PC 2 | PC 3 |
| --- | --- | --- | --- | --- |
| Eigenvalue |  | 6.41 | 4.33 | 1.25 |
| % variation |  | 42.76 | 28.90 | 8.33 |
|  |  |  |  |  |
| Trait | Abbeviation | PC 1 | PC 2 | PC 3 |
| C cell^-1^ | POC | 12.21 | 3.33 | 2.10 |
| N cell^-1^ | PON | 12.44 | 3.05 | 2.32 |
| P cell^-1^ | POP | 14.10 | 1.11 | 1.62 |
| Chl *a* cell^-1^ | Chl | 11.80 | 2.43 | 2.30 |
| Cell mL^-1^ | Density | 5.43 | 2.44 | 29.64 |
| Maximum fluorescence | Fmax | 5.58 | 7.05 | 0.01 |
| Growth rate (µ day^-1^) | gr | 1.70 | 4.04 | 20.82 |
| C:N ratio | CN | 2.48 | 5.97 | 2.88 |
| C:P ratio | CP | 0.12 | 21.77 | 0.38 |
| N:P ratio | NP | 0.52 | 12.18 | 0.47 |
| Chl : POC ratio | TchlPOC | 5.17 | 0.41 | 33.02 |
| N uptake | NO3up | 13.88 | 0.04 | 0.41 |
| P uptake | PO4up | 11.01 | 0.27 | 0.31 |
| RUE_N_ | RUEN | 3.30 | 14.08 | 3.73 |
| RUE_P_ | RUEP | 0.25 | 21.82 | 0.00 |

**Table S2**. PCA results of the 15 traits measured from all salinity conditions (0, 5, 15, 20, 30, and 35 psu).

|  |  | PC 1 | PC 2 | PC 3 |
| --- | --- | --- | --- | --- |
| Eigenvalue |  | 5.16 | 2.35 | 1.56 |
| % variation |  | 34.40 | 15.66 | 10.40 |
|  |  |  |  |  |
| Trait | Abbeviation | PC 1 | PC 2 | PC 3 |
| C cell^-1^ | POC | 0.05 | 26.72 | 0.11 |
| N cell^-1^ | PON | 1.32 | 25.41 | 3.37 |
| P cell^-1^ | POP | 1.73 | 27.30 | 2.08 |
| Chl *a* cell^-1^ | Chl | 0.17 | 4.72 | 18.60 |
| Cell mL^-1^ | Density | 10.09 | 0.68 | 4.17 |
| Maximum fluorescence | Fmax | 11.80 | 0.57 | 3.73 |
| Growth rate (µ day^-1^) | gr | 8.22 | 0.42 | 6.05 |
| C:N ratio | CN | 10.07 | 6.37 | 1.28 |
| C:P ratio | CP | 14.80 | 1.46 | 0.08 |
| N:P ratio | NP | 3.50 | 1.44 | 0.97 |
| Chl : POC ratio | TchlPOC | 5.89 | 2.34 | 3.55 |
| N uptake | NO3up | 0.24 | 0.04 | 29.30 |
| P uptake | PO4up | 0.59 | 0.14 | 23.14 |
| RUE_N_ | RUEN | 16.31 | 0.72 | 1.61 |
| RUE_P_ | RUEP | 15.22 | 1.59 | 1.96 |
